# Case Report: Hepatitis and autoimmune hemolytic anemia induced by EBV-associated infectious mononucleosis

**DOI:** 10.3389/fgstr.2026.1720586

**Published:** 2026-04-17

**Authors:** Akram Alnounou, Henry Zou, Madison C. Laird, Sophie Numbers, Christopher Visak, Sage Bilsland, Neil Hughes, Eric Martin Sieloff

**Affiliations:** 1 Department of Internal Medicine, Western Michigan University Homer Stryker MD School of Medicine, Kalamazoo, MI, United States; 2 Division of Gastroenterology, Bronson Methodist Hospital, Kalamazoo, MI, United States

**Keywords:** Epstein-Barr virus, hemolytic anemia, hepatitis, infectious mononucleosis, jaundice

## Abstract

Epstein-Barr virus (EBV) typically causes infectious mononucleosis, but in rare cases, it may lead to complications such as autoimmune hemolytic anemia (AIHA) and hepatitis. We report the case of an 18-year-old previously healthy female who presented with jaundice, red urine, and arthralgia. Laboratory workup revealed direct hyperbilirubinemia, elevated transaminases, and a direct antiglobulin test positive for IgG (negative for C3d), consistent with warm autoimmune hemolytic anemia in the context of EBV infection. Imaging ruled out biliary obstruction. The patient was managed conservatively, with cautious steroid use, and demonstrated clinical improvement without progression to hepatic failure. This case underscores the importance of recognizing atypical hepatic presentations of EBV and carefully managing overlapping autoimmune complications.

## Introduction

Epstein-Barr virus (EBV) is a common double-stranded DNA herpesvirus that establishes lifelong latency and infects more than 90% of people worldwide by the time they reach adulthood (1). In adolescents and young adults, primary infection often presents as infectious mononucleosis (IM), which classically causes fever, pharyngitis, and lymphadenopathy (2, 3). Hepatic involvement is frequent, with mild transaminase elevations seen in the majority of cases, but clinically significant EBV hepatitis is uncommon, occurring in fewer than 10% of cases (4, 5). Rarely, EBV triggers autoimmune hemolytic anemia (AIHA), a complication typically associated with immunocompromised hosts but also reported in healthy adolescents and young adults (5, 6). We present a case of EBV-induced AIHA and hepatitis in a previously healthy young adult.

## Case description

**Figure 1:**
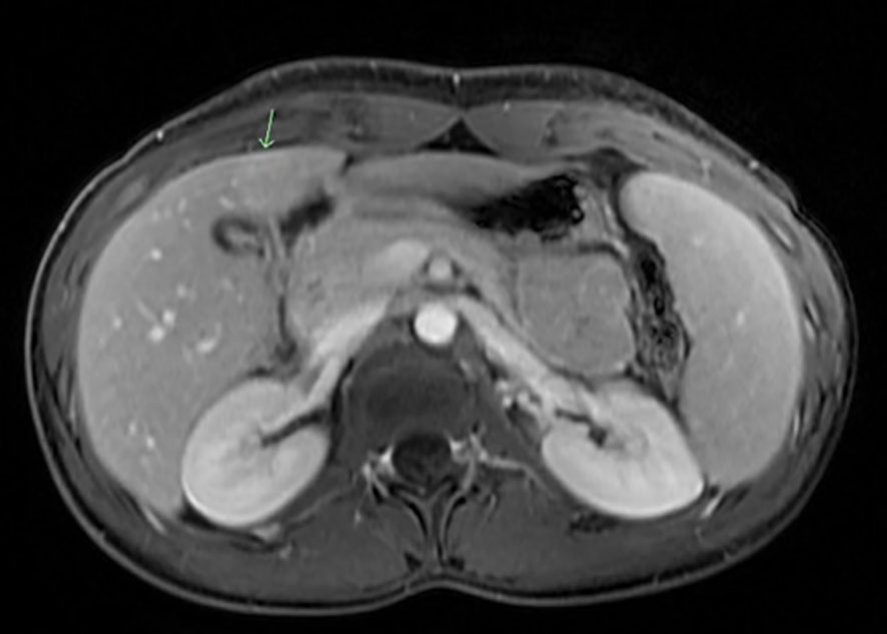
MRCP imaging showing focal nodular hyperplasia (green arrow). MRCP image demonstrating focal nodular hyperplasia appearing as a well-circumscribed hepatic lesion indicated by the arrow.

An 18-year-old female with no significant past medical history presented to the emergency department (ED) with abdominal pain, red urine, and generalized jaundice for the past day. She also endorsed arthralgias of her distal fingers, knuckles, and toes for the preceding four days. Her family history was noncontributory for liver disease, autoimmune conditions, or hematologic disorders. She had no surgical history, no known drug allergies, and denied tobacco, alcohol, or illicit drug use. Her only home medication was oral contraceptive norgestrel 75 micrograms daily. On examination, vital signs included a temperature of 37.4 °C, heart rate of 92 bpm, blood pressure of 118/72 mmHg, respiratory rate of 16 breaths/min, and oxygen saturation of 99% on room air. Physical examination revealed generalized jaundice and scleral icterus. The oropharynx was clear without pharyngitis or tonsillar enlargement. There was no cervical lymphadenopathy. The abdomen was soft with mild right upper quadrant tenderness but without hepatosplenomegaly. No rash was noted on admission. Her labs were notable for a total bilirubin of 11.5 mg/dL with direct hyperbilirubinemia (8.3 mg/dL) and elevated indirect bilirubin (3.2 mg/dL), elevated aspartate aminotransferase (215 U/L), and alanine aminotransferase (317 U/L). Lactate dehydrogenase (LDH) was 580 U/L (normal 120–246), haptoglobin was <8 mg/dL (normal 30–200), and reticulocyte count was 4.2% (normal 0.5–2.0%). Her hepatitis panel (for hepatitis A,B, and C), acetaminophen level, and gallbladder ultrasound results were unremarkable. Following hospital admission, an extensive laboratory workup for autoimmune (antinuclear antibody, anti-smooth muscle antibody, anti-mitochondrial M2 antibody, liver-kidney microsome antibody, inflammatory bowel disease [IBD] serology panel), infectious (human immunodeficiency virus [HIV], cytomegalovirus [CMV], parvovirus B19), and hereditary etiologies (ceruloplasmin levels) was unremarkable except for her IM screen, EBV immunoglobulin M (IgM), and heterophile antibodies, which were positive. Monospecific direct antiglobulin testing (DAT) was positive for IgG and negative for complement (C3d). Hemoglobin decreased from 11.2 to 8.9 g/dL during hospitalization days 1–4. Magnetic resonance cholangiopancreatography (MRCP) revealed gallbladder wall edema and focal nodular hyperplasia (FNH) of the liver (**Figure 1**).

**Figure 2:**
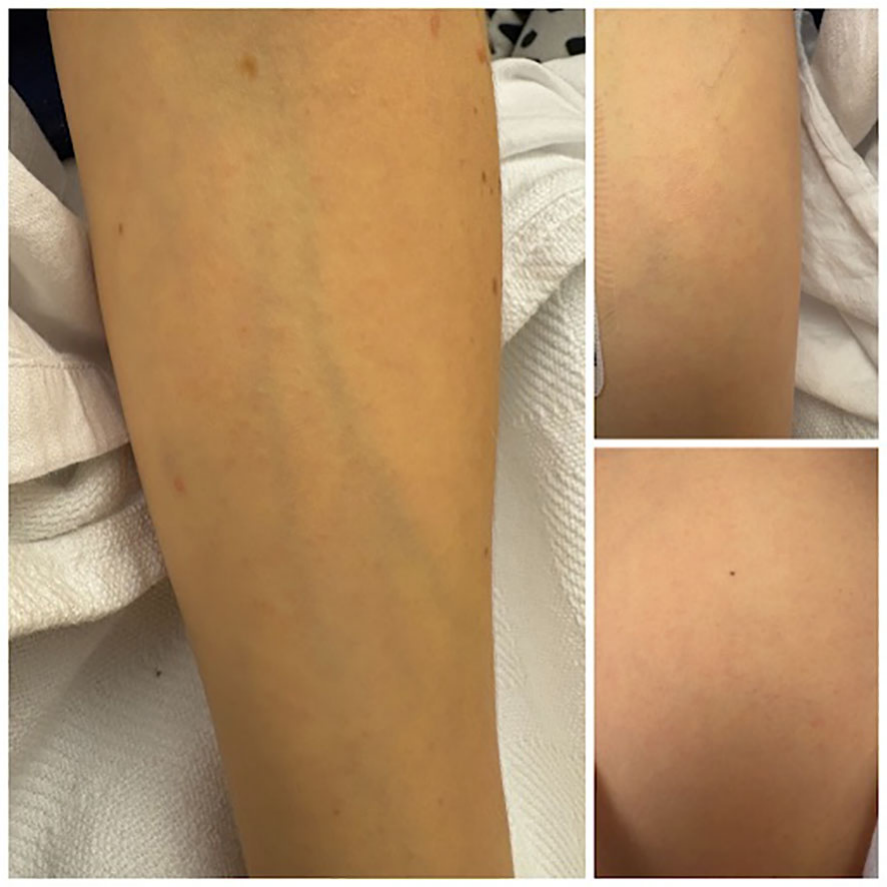
Non-blanching erythematous papules on the patient’s bilateral forearms and right thigh. Clinical photograph showing multiple scattered non-blanching erythematous papules on the forearms and thigh.

Gastroenterology and Hematology/Oncology were consulted, and she underwent supportive care (hydration and intravenous fluid repletion) with close monitoring of her clinical status. A trial of prednisone 40 mg daily for AIHA control was discontinued after two doses due to concerns about immunosuppression. Over the next three days, the patient’s bilirubin, AST, and ALT steadily decreased, while her hemoglobin gradually increased from 8.9 to 9.3 g/dL after initial fluctuation. Concurrently, LDH declined from 580 to 285 U/L, reticulocyte count rose to 5.8%, and haptoglobin increased to 15 mg/dL by discharge. However, she developed a non-pruritic, non-painful rash of her bilateral forearms and right thigh consisting of scattered, discrete, non-blanching red papules (**Figure 2**).

She was discharged after five inpatient days on topical triamcinolone 0.1% and hydrocortisone 1% for her rash and a reduced dose of her home oral contraceptive norgestrel (from 75 to 30 micrograms) given her hepatitis and FNH. A detailed timeline of the patient’s clinical course, including symptoms, laboratory findings, imaging, treatments, and follow-up, is presented in **Table 1**.

**Table 1:** Timeline of clinical presentation, diagnostic evaluation, therapeutic interventions, and outcomes.

Day	Clinical events	Hb (g/dL)	Total Bili (mg/dL)	Direct Bili (mg/dL)	Indirect Bili (mg/dL)	AST/ALT (U/L)	ALP/GGT (U/L)	LDH (U/L) [N: 120–246]	Haptoglobin (mg/dL) [N: 30–200]	Reticulocyte (%) [N: 0.5–2.0]	EBV PCR (copies/mL)	Interventions & notes
–4	Onset of polyarthralgia (distal fingers, knuckles, toes)	—	—	—	—	—	—	—	—	—	—	Symptoms began at home
–1	Jaundice, red urine, abdominal pain	—	—	—	—	—	—	—	—	—	—	Presented to ED
0 (Admit)	Hospitalized with jaundice and arthralgia. DAT: IgG positive, C3d negative → warm AIHA. EBV IgM+, heterophile+, IM screen+. Hepatitis A/B/C, HIV, CMV, parvovirus B19: negative. ANA, anti-smooth muscle Ab, anti-mitochondrial M2 Ab, liver kidney microsome Ab, IBD panel: negative. Ceruloplasmin: normal. US: unremarkable gallbladder. MRCP: GB wall edema, focal nodular hyperplasia.	11.2	11.5	8.3	3.2	215/317	222/183	580	<8	4.2	18,400	IV fluids initiated. GI and Heme/Onc consulted.
1	EBV infection confirmed; clinical stability maintained	10.5	—	—	—	—	—	—	—	—	—	Prednisone 40 mg daily started for warm AIHA
2	Non-pruritic, non-painful rash: scattered, discrete, non-blanching red papules on bilateral forearms and right thigh	9.8	—	—	—	—	—	—	—	—	—	Prednisone discontinued after 2 doses (immunosuppression concern with active EBV hepatitis). Topical triamcinolone 0.1% and hydrocortisone 1% started for rash.
3	Liver enzymes and bilirubin improving; no new symptoms	9.1	6.1	4.2	1.9	150/180	—	390	—	—	8,600	Continued supportive care. EBV PCR declining; steroids not reinitiated.
4	Hemoglobin nadir; clinical stability maintained	8.9	—	—	—	—	—	—	—	—	—	Close monitoring. No transfusion required.
5 (Discharge)	Clinically stable; labs trending toward normalization	9.3	3.8	2.7	1.1	—	—	285	15	5.8	3,100	Discharged on topical corticosteroids. Norgestrel reduced 75→30 mcg (hepatitis and FNH). Outpatient follow-up recommended.

Hb, hemoglobin; Bili, bilirubin; AST, aspartate aminotransferase; ALT, alanine aminotransferase; ALP, alkaline phosphatase; GGT, gamma-glutamyl transferase; LDH, lactate dehydrogenase; DAT, direct antiglobulin test; AIHA, autoimmune hemolytic anemia; EBV, Epstein-Barr virus; IM, infectious mononucleosis; PCR, polymerase chain reaction; MRCP, magnetic resonance cholangiopancreatography; GB, gallbladder; FNH, focal nodular hyperplasia; GI, gastroenterology; Heme/Onc, hematology/oncology; IV, intravenous; US, ultrasound; ANA, antinuclear antibody; Ab, antibody; IBD, inflammatory bowel disease; N, normal range. A dash (—) indicates a value not obtained or not applicable at that time point.

This table outlines the key events from symptom onset through hospital discharge and follow-up, including laboratory results, diagnostic procedures, treatment decisions, and clinical progression of the patient’s Epstein-Barr virus–associated autoimmune hemolytic anemia and cholestatic hepatitis.

## Diagnostic assessment, therapeutic intervention, follow-up, and outcomes

Our diagnostic workup was deliberately broad, given the overlap of symptoms. Initial laboratory findings were consistent with concurrent cholestatic hepatitis and hemolytic anemia, with a positive DAT (IgG-positive, C3d-negative). Viral screening narrowed the etiology to acute EBV infection, supported by positive heterophile and EBV IgM antibodies. Autoimmune markers and other infectious serologies were negative, and imaging studies—including ultrasound and MRCP—excluded biliary obstruction or other structural causes. This comprehensive approach allowed us to rule out alternative diagnoses such as autoimmune hepatitis or drug-induced liver injury and avoid invasive procedures such as liver biopsy.

The patient was managed primarily with supportive care, including IV fluids and symptom control. Given the diagnosis of AIHA, corticosteroid therapy (prednisone 40 mg daily) was initiated but halted after two doses due to concerns that immunosuppression might worsen her viral hepatitis. Close monitoring of liver function tests and hemoglobin levels guided this decision. Over the subsequent days, the patient’s liver enzymes and bilirubin steadily improved, and hemoglobin stabilized without further steroids. EBV viral load by PCR was monitored longitudinally to guide immunosuppressive therapy: levels declined from 18,400 copies/mL at admission to 8,600 copies/mL by Day 3 and 3,100 copies/mL at discharge, confirming active viral clearance and supporting the decision not to reinitiate corticosteroids. Topical corticosteroids were prescribed to address the rash that emerged during recovery, and her oral contraceptive norgestrel dose was reduced (from 75 to 30 micrograms), given the acute hepatitis and impaired hepatic metabolic capacity.

After five days, the patient was discharged with improved clinical and biochemical markers. Follow-up at the outpatient clinic was recommended to confirm continued recovery with no relapse of hemolysis or hepatic dysfunction.

## Discussion

This case highlights a rare and clinically challenging presentation of concomitant EBV-induced cholestatic hepatitis and AIHA in an otherwise healthy 18-year-old woman. Although EBV-induced cholestatic hepatitis and AIHA have been individually described in the literature, their simultaneous presentation is exceedingly rare (7, 8).

The hemolytic process was confirmed by a combination of suppressed haptoglobin (<8 mg/dL), elevated LDH (580 U/L), and reticulocytosis (4.2%), a constellation that has been shown to be highly specific for hemolysis (9). Longitudinal monitoring demonstrated resolving hemolytic activity: LDH declined from 580 to 285 U/L, the reticulocyte count rose from 4.2% to 5.8%, reflecting appropriate marrow compensation, and haptoglobin recovered from <8 to 15 mg/dL by discharge. Of note, the concurrent hepatocellular injury likely contributed to the LDH elevation, an important consideration when interpreting this marker in presentations with dual pathology. Bilirubin fractionation further clarified the respective contributions to jaundice: direct hyperbilirubinemia (8.3 mg/dL) predominated, consistent with cholestatic hepatitis as the primary driver, while the concurrent indirect bilirubin elevation (3.2 mg/dL) reflected the hemolytic component. Both fractions improved during hospitalization—total bilirubin declined from 11.5 to 3.8 mg/dL—with the indirect component normalizing faster than the direct, consistent with more rapid resolution of hemolysis relative to hepatic recovery. This pattern is consistent with the observation that jaundice in EBV-related AIHA with hepatic involvement reflects contributions from both hemolysis and hepatic dysfunction (6).

Notably, monospecific DAT in our patient was positive for IgG and negative for C3d, classifying this as warm AIHA. This finding is atypical for EBV-associated AIHA, which is more commonly mediated by cold agglutinins (IgM antibodies directed against I/i erythrocyte antigens) via molecular mimicry and complement activation (6, 10, 11). In a recent literature review of 17 cases of EBV-related AIHA, 76% were cold-mediated, with only a single case classified as warm AIHA (6). However, the First International Consensus on AIHA recognizes that the immunoglobulin class in EBV-associated hemolysis can be either IgG or IgM, and EBV is listed among infections associated with secondary warm AIHA (9). The mechanism of warm AIHA in EBV likely involves polyclonal B-cell activation producing IgG autoantibodies against erythrocyte surface antigens such as Rh epitopes, rather than the molecular mimicry–driven cold agglutinin pathway (11). This distinction carries direct therapeutic implications, as the management of warm and cold AIHA differs fundamentally (9, 10) and is discussed further below. Prognostically, EBV-associated AIHA in immunocompetent patients is generally self-limiting, with favorable outcomes reported across all published cases in which outcome was documented (6). Nevertheless, close monitoring was warranted in our patient, given the concurrent hepatitis and its potential for fulminant decompensation (5).

Our patient initially presented with polyarthralgia, jaundice, dark urine, and a cholestatic biochemical profile, necessitating a broad differential diagnosis that included biliary obstruction, autoimmune hepatitis, and drug-induced liver injury. Our comprehensive diagnostic approach encompassed autoimmune (autoimmune hepatitis, primary biliary cholangitis, and IBD), hereditary (Wilson disease, Gilbert’s syndrome, and Rotor syndrome), and infectious (hepatitis A, B, and C, HIV, CMV, and parvovirus B19) etiologies. Her cholestatic clinical presentation, characterized by direct hyperbilirubinemia (8.3 mg/dL), elevated gamma-glutamyl transferase (183 U/L), and alkaline phosphatase (222 U/L), guided us toward to perform ultrasound followed by MRCP to exclude intrahepatic and extrahepatic cholestasis. This systematic strategy of laboratory and imaging evaluations enabled us to avoid invasive procedures such as liver biopsy and prevent misdiagnosis of a condition that could rapidly decompensate into liver failure. This case demonstrates how atypical presentations of EBV infections can closely mimic a range of hepatobiliary conditions; therefore, assessing for a broad differential diagnosis while recognizing this wider clinical spectrum of EBV infection is crucial.

Balancing the management of AIHA with the risk of exacerbating EBV hepatitis posed a unique challenge in our patient. Corticosteroids are the established first-line therapy for warm AIHA, with approximately 80% of patients responding to doses equivalent to prednisone 1 mg/kg daily (9). Given the confirmed warm AIHA subtype in our patient, the decision to initiate prednisone 40 mg daily was appropriate and consistent with current consensus recommendations (9). Had this been cold agglutinin–mediated hemolysis, corticosteroids would not have been indicated, as they are ineffective in cold agglutinin disease and B-cell–directed therapy such as rituximab would be preferred (9, 10). However, in the context of concurrent EBV hepatitis, immunosuppression posed a risk of promoting uncontrolled viral replication and exacerbating hepatic inflammation (4, 12). Prednisone was therefore discontinued after two doses, prioritizing hepatic safety. EBV PCR levels were monitored longitudinally to inform the timing of potential steroid reinitiation: viral load declined from 18,400 copies/mL at admission to 8,600 copies/mL by Day 3 and 3,100 copies/mL by discharge, an 83% reduction indicating effective viral clearance without sustained immunosuppression. This objective virological improvement, combined with stabilizing hemoglobin and declining liver enzymes, confirmed that further corticosteroids were unnecessary. The self-limited course is consistent with the natural history of infection-associated secondary AIHA, which typically resolves spontaneously as the inciting infection clears (9, 10). This case illustrates how precise AIHA subtyping informs the therapeutic rationale: the confirmation of warm AIHA justified the initial corticosteroid trial, while the concurrent EBV hepatitis appropriately limited its duration. In clinical scenarios where the AIHA subtype is not determined, therapeutic decision-making is compromised, particularly in EBV-associated presentations where the cold versus warm distinction dictates fundamentally different management strategies. Our avoidance of immunosuppressive agents mitigated the risk of viral hepatitis exacerbation that could progress to liver failure. This conservative strategy parallels another case of a 15-year-old female with EBV-induced cholestatic hepatitis and AIHA who recovered after three weeks of inpatient supportive care (7). However, it contrasts with the case of a 16-year-old male with EBV-induced hepatitis and AIHA who received a prednisolone course and recovered after 12 days of hospitalization (7, 8). Our patient’s oral contraceptive norgestrel dose was reduced (from 75 to 30 micrograms) at discharge. This decision was driven primarily by the acute hepatitis, as oral contraceptives undergo extensive hepatic first-pass metabolism, and dose reduction is prudent during active hepatic inflammation with impaired metabolic capacity. Notably, current EASL guidelines indicate that FNH alone does not require discontinuation or modification of oral contraceptive use, as hormonal contraceptives have not been demonstrated to play a role in FNH development or progression (13). In our patient, the combination of active hepatitis with a newly identified hepatic lesion warranted a conservative approach to minimize additional hepatic metabolic burden during recovery.

This case highlights the importance for clinicians to consider atypical presentations of EBV in their differential diagnoses to facilitate proper management of a potentially acute condition and reduce invasive testing. Furthermore, it illustrates the necessity of balancing the management of autoimmune and infectious etiologies to avoid acute exacerbations of either condition.

## Strengths and limitations

This case has several strengths. The comprehensive diagnostic workup, encompassing autoimmune, hereditary, and infectious etiologies, enabled a definitive diagnosis while avoiding invasive procedures such as liver biopsy. The multidisciplinary involvement of gastroenterology and hematology/oncology facilitated individualized management of competing pathological processes. Additionally, monospecific DAT testing allowed classification of the AIHA subtype, informing the therapeutic rationale.

Several limitations should be acknowledged. Certain hemolysis markers, including serial haptoglobin values and peripheral blood smear review, were not obtained at all time points during hospitalization, limiting the granularity of hemolytic activity assessment. Cold agglutinin titers were not formally tested, although the DAT profile (IgG-positive, C3d-negative) and clinical presentation were consistent with warm AIHA rather than cold agglutinin disease. Long-term follow-up data beyond discharge are not yet available, and the possibility of delayed relapse of hemolysis or hepatic dysfunction cannot be excluded. Finally, as a single-case report, the findings are inherently limited in generalizability but nonetheless contribute to the small body of literature on this rare dual presentation.

## Patient perspective

The patient was informed throughout her hospital stay and involved in decisions regarding her care, particularly the cautious use of steroids. The patient’s father expressed relief at the steady improvement in the patient’s symptoms and appreciated the multidisciplinary team’s attentiveness.

## Data Availability

The original contributions presented in the study are included in the article/supplementary material. Further inquiries can be directed to the corresponding author.
